# Semantic encoding of trauma memories in the hippocampus among individuals with PTSD

**DOI:** 10.1038/s41386-026-02402-5

**Published:** 2026-04-14

**Authors:** Josh M. Cisler, Luna T. Malloy, Michael Jaeb, Joseph E. Dunsmoor, Zachary N. Stowe

**Affiliations:** 1 Department of Psychiatry and Behavioral Sciences, Dell Medical School, University of Texas at Austin, Austin, TX USA; 2 Institute for Early Life Adversity Research, Dell Medical School, University of Texas at Austin, Austin, TX USA; 3 Department of Psychiatry, University of Wisconsin at Madison, Madison, WI USA; 4 Department of Neuroscience, University of Texas at Austin, Austin, TX USA

**Keywords:** Trauma, Post-traumatic stress disorder

## Abstract

The recall of traumatic memories is central to clinical and neurobiological models of PTSD, yet neurocircuitry mechanisms underlying traumatic memory recall remain elusive. Recent advances in natural language processing and large language models enable complex semantic quantification of autobiographical memories. Here, we leveraged these analytic approaches to define the neurocircuitry encoding the semantic content of traumatic autobiographical narratives among individuals with PTSD. 79 women with PTSD related to interpersonal violence listened to traumatic and neutral autobiographical narratives during fMRI. Sentence-level brain activity and semantic embeddings were quantified for each script and participant. Neurocircuitry encoding semantic content of the narratives was defined through cross-validation across participants. A priori regions of interest included the hippocampus, superior temporal gyrus (STG), and posterior cingulate cortex (PCC). Our approach detected significant hippocampal sensitivity for semantic content of both trauma and neutral narratives; however, spatial encoding patterns of semantic content within the hippocampus differed between trauma and neutral narratives. Specifically, spatial encoding patterns in CA1 and dentate gyrus differentiated narrative type. Regardless of narrative type, PTSD symptom severity was positively associated with semantic encoding across the hippocampus and its subfields, except for the subiculum. For trauma narratives, semantic sensitivity was greater within the left STG and decreased in the PCC and broader default mode network. Encoding in neither region tracked with PTSD symptom severity. These results reveal a hippocampal role in mediating recall of specific semantic content for traumatic and neutral autobiographical narratives and suggest hippocampal sensitivity to autobiographical semantic content underlies greater PTSD symptom severity. Clinical trial registration information: Improving Therapeutic Learning for PTSD, Study Details | NCT04558112 | Improving Therapeutic Learning for PTSD | ClinicalTrials.gov, NCT04558112.

## Introduction

Posttraumatic Stress Disorder (PTSD) is a mental health disorder with significant morbidity, comorbidity, and decreased quality of life [1, 2]. Memory of the traumatic event is central to most theories of PTSD [3] and forms the basis for understanding many clinical signs and symptoms [4–7], including intrusive recollections and nightmares of the trauma, flashbacks, avoidance of internal and external stimuli that may trigger trauma-related memories, and traumatic beliefs. Despite widespread agreement on the centrality of the trauma memory for explaining PTSD symptoms, the nature of these memory representations, and whether there are distinct neurocircuitry mechanisms supporting trauma and normative autobiographical memory, has remained elusive [4]. In particular, the role of the hippocampus in mediating recall of the traumatic memory in PTSD has been inconsistently defined across studies, with meta-analyses generally failing to detect hippocampal activation during trauma memory recall [8, 9], some studies identifying differential patterns of connectivity with the hippocampus during trauma memory recall [10, 11] and other studies not finding differential hippocampal connectivity [12], and prominent theoretical accounts arguing the trauma memory is unique for its lack of hippocampal involvement [13]. The inconsistent definition of a role for the hippocampus in trauma memory recall is striking, given strong evidence for hippocampal involvement in the retrieval of autobiographical memory [14–16] as well as its prominent role in neurocircuitry models of PTSD informed by both basic and clinical research [17–20]. Here, we apply a novel analytical and methodological approach that addresses key limitations of prior work to rigorously test hypotheses about hippocampal contributions to traumatic memory recall in PTSD.

An important feature of so-called ‘traumatic memory’ in PTSD is that it rarely exists as a unitary representation [21]. Rather, what is typically referred to as the ‘traumatic memory’ is better understood as a traumatic *narrative*, involving a complex constellation of distinct events, details, and sensory fragments that encompass an individual’s recollection. Recognizing this complexity has important clinical, methodological, and theoretical implications. Clinically, exposure-based therapies often focus on the most distressing segments of a narrative, referred to as “hot spots” [22–25], to maximize extinction learning and symptom reduction. But these hot spots are embedded within a broader network of memories that contribute the contextual, temporal, and sensory details that elicit comparatively less distress on their own. Accordingly, contemporary therapies have adapted to the heterogeneity of traumatic narratives to address both the emotionally charged and less distressing elements of memory [25, 26].

Methodologically, acknowledging that a traumatic memory is in fact a complex traumatic narrative is consequential for how it is studied. Investigating the cognitive, emotional, and neurocircuitry processes underlying traumatic recall requires careful delineation of distinct moments and details within the narrative. If these elements are collapsed together—for example, averaging neural activity across an entire memory in neuroimaging research—critical associations between specific memory elements and corresponding neural processes are obfuscated. Hence, treating the memory as a unitary representation rather than a narrative has empirical risks for blurring fine-grained processes and testing theoretical models of traumatic memory recall. Although many investigations have examined cognitive, neurocircuitry, and psychophysiological responses during recall of traumatic narratives [12, 27–35], and despite acknowledgement of the complexity of trauma narratives [21, 36], there have been limited neuroimaging efforts to systematically account for the heterogeneity inherent to traumatic narratives [10, 37]. These methodological challenges accordingly hinder theoretical inferences. For example, linking hippocampal findings in PTSD populations to more basic theories of hippocampally-mediated memory that predict overlap between distinct memory types [38, 39] is difficult when methodological factors obscure more fine-grained analyses of trauma narratives.

An emerging approach is to apply contemporary natural language processing methods, which are increasingly sophisticated in quantifying the semantic content of natural language, such as that expressed in trauma narratives. Among these, sentence transformers [40] have become a dominant architecture for generating semantic embeddings, high-dimensional vector representations of meaning that capture context-dependent relationships among words. Unlike earlier models that represented words in isolation [41], sentence transformers can encode phrases and sentences as unified embeddings to provide a richer representation of contextually-dependent meaning. A recent study [13] used semantic embeddings to examine neurocircuitry underlying trauma memory using a word-based model (word2vec [41]), and appeared to demonstrate that the semantic content of trauma memory was not represented in the hippocampus, but rather the posterior cingulate cortex. In this work, embeddings were generated for each word in a trauma narrative, then averaged within each sentence and then averaged again across sentences to derive a single representation of the narrative. Brain activity was measured in similarly broad manner that involved averaging the BOLD response across an entire script, rather than event-specific beta coefficients. While this study employed innovative methods, there were nonetheless key limitations. For example, averaging semantic embeddings of each word across the entire narrative obscures the complexity of the trauma narrative. Similarly, averaging brain activation across the entire narrative obscures detection of differential brain activation to unique components of the trauma narrative. Finally, the word-based semantic embedding approach does not adequately capture the semantic meaning of words across phrases and sentences. Accordingly, the conclusion that the hippocampus does not encode or respond to semantic content of trauma memories [13], and the broader claim that trauma memories constitute a unique class of cognitive entities distinct from other emotional memories [27], should be interpreted with caution.

Here, we build on this prior study and longer tradition of studying the traumatic memory in PTSD to focus specifically on a finer-grained and moment-by-moment analysis of trauma narratives that mirrors contemporary methodology examining neurocircuitry encoding of natural language [42–44]. We use a recent and high performing sentence transformer [45] to quantify unique semantic embeddings for each sentence in a trauma and neutral narrative among individuals with PTSD, and in parallel quantify brain activity specific to each sentence within these narratives. This affords the ability to capture how the hippocampus responds to semantic content of an autobiographical narrative on a sentence-by-sentence basis. Our approach is nested within individuals, which also allows us to characterize how individual differences in PTSD symptoms might modulate the neurocircuitry encoding semantic content during recall of trauma and neutral narratives. Consistent with other studies demonstrating hippocampal encoding of semantic information [46, 47], and consistent with a prominent role of the hippocampus in PTSD [20, 48, 49] and normative autobiographical memory [14–16, 50], we hypothesize that the hippocampus encodes semantic details of both traumatic and neutral narratives, thereby failing to support the alternative hypothesis that trauma memories are not processed semantically in PTSD [13, 27]. We also include the left superior temporal gyrus (i.e., Wernecke’s area) and posterior cingulate cortex as regions of interest given their canonical roles in language and self-directed mentation, respectively [51–53]. The hippocampus is not canonically associated with language or semantic encoding of language, though it certainly has roles in these cognitive processes [47, 52]. Accordingly, these additional regions of interest bolster tests of the hypothesis that trauma narratives are recalled differently from other autobiographical narratives beyond just hippocampal processes. Finally, we also differentiate between subfields of the hippocampus, given subfield specificity regarding hippocampal processes relevant to PTSD [54–57].

## Methods

### Participants

Participants consisted of 79 adult women, aged 21–50, with a current diagnosis of PTSD related to interpersonal violence, collected at two different sites (University of Wisconsin, *n* = 41; University of Texas at Austin, *n* = 38). One additional participant was excluded due to excessive head motion. Supplemental Table 1 lists demographic and clinical characteristics. All procedures were approved by the appropriate IRBs and all participants provided informed consent.

### Autobiographical narrative task

The task used here was modeled after our prior study [11], which is an adaptation of commonly used script-driven imagery tasks [29–31, 58]. Two scripts, one detailing a minimally arousing and emotionally neutral event and one detailing the individual’s index traumatic event, were created through a collaborative interview between participants and a research staff member. Scripts were completed with research staff, rather than self-guided by the participant, to ensure comparability across participants in detail and length and prevent avoidance, with narrative length constrained to 330-350 words. More task details are provided in the supplement.

During scanning, audio recordings of each script were accompanied by images of the text of the script displayed in white font against a black background. This allowed the participant to read along as they listened to their memory. Each narrative was presented four times in a row, with the neutral narrative always occurring first. Participants provided ratings of anxiety, vividness, and dissociation after each narrative repetition. Supplemental Fig. 1 displays the average subjective ratings for each repetition. One important caveat about this methodology is the fixed order: four neutral script repetitions followed by four trauma script repetitions. As such, possible habituation effects could confound script type differences.

### MRI procedures

See Supplementary Methods for information on neuroimaging parameters and preprocessing.

### Semantic neurocircuitry encoding models

**Fig. 1:**
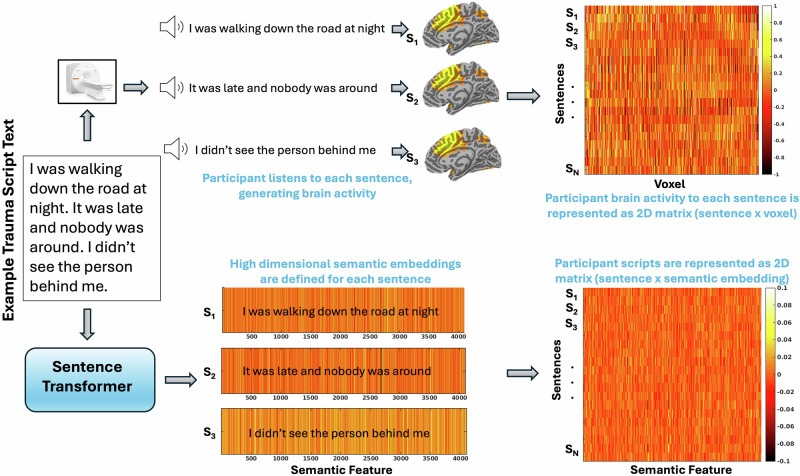
Depiction of within-participant methodology for extracting semantic features and brain activity for each sentence from an autobiographical narrative. The timing and duration of each sentence within a narrative script is calculated and used to build an LSS design matrix, resulting in voxelwise beta coefficients of activity unique to each sentence. In parallel, a sentence transformer processes each sentence and provides a high-dimensional semantic embedding that quantifies the latent semantic meaning of the sentence. The sentence x voxelwise beta coefficients, and the sentence x semantic embeddings, are represented by 2D matrices for each participant.

Our approach follows similar methodology of studies investigating neurocircuitry of semantic encoding [42–44]. An initial step was within-subject quantification of brain activity and semantic embeddings for each script (Fig. 1). Voxelwise brain activity, within a study-specific gray matter mask, was defined for every sentence within each script using GLMs implemented within a standard MVPA LSS framework (AFNIs 3dLSS). The time and duration of each sentence was extracted automatically from each audio file using the Whisper Python library [59]. Semantic embeddings were extracted using the NV-Embed-V2 [45] sentence transformer, a recent model with high performance relative to other sentence transformers [60], providing 4096 features for each sentence. Accordingly, brain activity for each participant was represented by a sentence x voxelwise activity matrix, and sentence semantic embeddings represented as sentence × embeddings matrix. These matrices were calculated for all participants and concatenated, resulting in a 3928 × 63,789 sentence × brain activity matrix, and 3928 × 4096 sentence x semantic embeddings matrix.

**Fig. 2:**
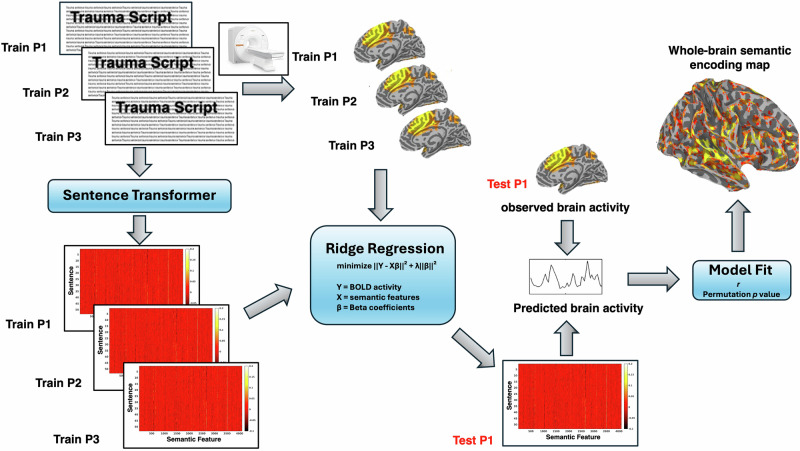
Depiction of methodology across subjects for building a model predicting brain activity to a given sentence based on semantic features of that sentence. A 10-fold cross-validation procedure is used, selecting a training set of participants and a left out set of test participants. The 2D sentence × voxelwise brain activity matrices, described in Fig. 1, are concatenated across training participants, as are the sentence x semantic embedding matrices. Separately for each voxel, ridge regression is then used to build a model predicting that voxel’s activity from the semantic features. The resulting model is then applied to the left out test set of participant’s semantic features, providing predicted voxel activity. The predicted voxel activity for each sentence for each participant is compared to the observed activity, providing an index of model fit.

The next step was training and testing group-level models of voxelwise semantic encoding (Fig. 2). Following prior research [42–44], ridge regression was used to predict brain activity to each sentence from the sentences’ semantic embeddings. A 10-fold cross-validation loop across participants was used. A separate model was trained and tested for each voxel for trauma narratives and for neutral narratives. Group-level model fit was calculated as the Pearson *r* of observed brain activity with predicted brain activity across the held-out test cases. *P* values for group-level semantic encoding fit were calculated using permutation testing with 1000 iterations [42]. Participant-level model fit was r-to-z transformed correspondence between observed and predicted activity calculated uniquely for each participant and script type.

Whole-brain analyses used cluster-level thresholding, achieving a corrected *p* < 0.05 through 21 contiguous voxels of *p* < 0.001 [61, 62]. Whole-brain LMEs testing associations between semantic encoding, script type, and PTSD symptoms used the following model: semantic encoding ~ script type × PTSD severity + age + education + site + medication + framewise displace + (script type | subject).

### Regions of interest analyses

The supplement describes methodology for defining the hippocampus, PCC, and STG ROIs and hippocampus subfields.

Semantic encoding in the hippocampus was analyzed as follows. First, mean activity within the bilateral hippocampus mask across participants was compared between script types and as a function of PTSD symptom severity (CAPS severity scores) with linear mixed effects models (LMEMs): semantic encoding ~ script type × PTSD severity + age + education + site + medication + framewise displace + (script type | subject). Analyses of the entire hippocampus were initially collapsed across both hemispheres [13]. Secondary analyses differentiated hippocampal subfields and included hemisphere as an additional interaction term: semantic encoding ~ script type × PTSD severity × hemisphere + age + education + site + medication + framewise displace + (script type | subject).

Third, the degree to which spatial patterns of semantic encoding within the hippocampal subfields differentiated script types was tested using support vector machine classifiers (SVCs). For these SVCs, voxels for each hemisphere were combined, as the LMEMs did not reveal a significant effect of hemisphere. The SVCs were trained and tested, using 10-fold cross-validation across participants, to differentiate trauma from neutral narratives based on spatial patterns of semantic encoding for voxels within a given ROI. To ensure the spatial pattern of semantic encoding, rather than the overall magnitude of semantic encoding, was driving SVC performance, the participant observation × voxel matrices were centered across voxels within a given observation prior to training the classifiers. Classification performance was defined as median area-under-the-curve of receiver operating characteristics across the 10 folds, with statistical significance determined through permutation testing with 10,000 iterations.

Semantic encoding of the PCC and left STG was analyzed using an LMEM in parallel to the initial hippocampus analyses: semantic encoding ~ script type × PTSD severity + age + education + site + medication + framewise displace + (script type | subject).

### Cross-narrative semantic encoding models

As an additional means of testing the hypothesis of differential semantic encoding for trauma vs neutral narratives, we also tested cross-narrative neurocircuitry semantic encoding models. These models were trained identically as described above, but were tested on the alternative narrative dataset. For example, once a model was built to predict a voxel’s activity given neutral semantic content, this model was then tested on voxel activation during a trauma narrative given traumatic semantic content. As such, these models stringently test whether voxels encode semantic content similarly for trauma and neutral narratives. Model fit for the cross-tested models was defined identically as described above.

### Sentence activity classification analyses

Finally, to elaborate the role of ROI activity during each sentence of the narratives, in addition to testing semantic encoding, we conducted additional classification analyses. These SVC analyses tested whether patterns of voxel activity to each sentence could differentiate narrative types; that is, these classification analyses did not consider semantic content of the sentences and only tested if patterns of sentence activity during the narrative could differentiate narrative type. 10-fold cross-validation across participants was used to train an SVC model to differentiate the trauma from neutral narratives given patterns of voxel activity in each ROI. Voxel activity patterns were centered within each sentence to ensure patterns of activity, rather than overall activity magnitude, contributed to the classifier. Classification performance was defined as median area-under-the-curve of receiver operating characteristics across the 10 folds, with statistical significance determined through permutation testing with 10,000 iterations.

## Results

### Semantic content differentiates trauma and neutral autobiographical narratives

As validation of the sentence transformer methodology, we examined similarity of semantic embeddings for trauma and neutral sentences (See Supplemental Material and Supplemental Fig. 2). Additionally, lexical properties of the narratives (word length, sentence length, number of sentences) did not differ between narrative types (see Supplemental Material).

### The hippocampus and its subfields respond to semantic content of autobiographical memories

**Fig. 3:**
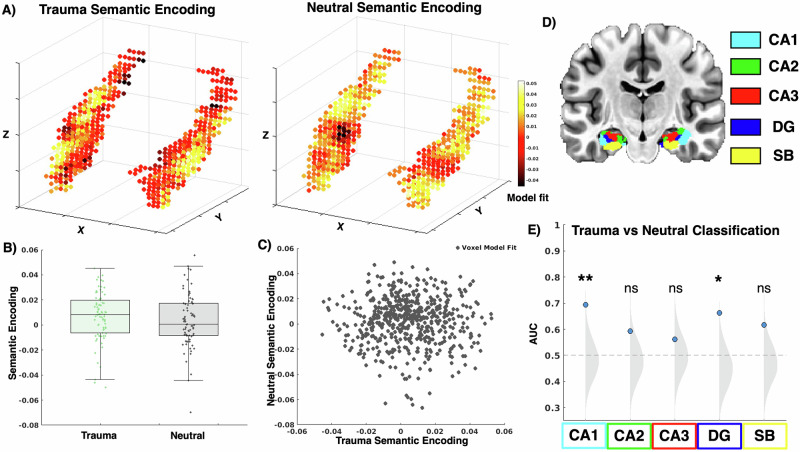
Semantic encoding in the hippocampus. **A** 3D representations of voxelwise semantic encoding in the left and right hippocampus. Each dot represents a voxel, with the color corresponding to the degree of semantic encoding; i.e., the degree to which brain activity to a given sentence can be predicted from semantic features for that sentence. **B** Means and distributions of hippocampal semantic encoding across participants for trauma and neutral narratives. **C** Scatter plot depicting a null relationship between semantic encoding for each voxel for trauma and neutral narratives. **D** Anatomical depiction of hippocampal subfields CA1, CA2, CA3, dentate gyrus (DG), and subiculum (SB). **E** Results of SVC classification analyses, testing if patterns of voxels’ semantic encoding differentiates trauma from neutral narratives. The Y-axis represents area-under-the-curve (AUC) for receiver operating characteristics. The blue dots represent the median AUC classification performance across a 10-fold cross-validation loop, with the dotted line depicting chance AUC classification performance. The gray distributions depict the permuted null distribution for that classification analysis across 10,000 iterations. ** = *p* < 0.001; * = *p* = 0.01, ns = not significant.

Across participants, mean hippocampal semantic encoding was significant, *t*(140) = 4.05, *p* < 0.001, with no difference between trauma and neutral memories, *p* = 0.27. The spatial pattern of hippocampal voxels’ semantic encoding is depicted in Fig. 3A. While the distribution of voxels’ semantic encoding overlapped for trauma and neutral memories (Fig. 3B), the spatial patterns of voxel’s semantic encoding across memory types were uncorrelated (Fig. 3C). To further test distinct spatial patterns of semantic encoding by narrative type, SVCs were trained (see methods) to differentiate narrative type from spatial patterns within distinct hippocampus subfields (Fig. 3D). Spatial patterns within CA1 (AUC = 0.69, *p* = 0.002) and the dentate gyrus (AUC = 0.66, *p* = 0.01) differentiated trauma vs neutral memories (Fig. 3E).

**Fig. 4:**
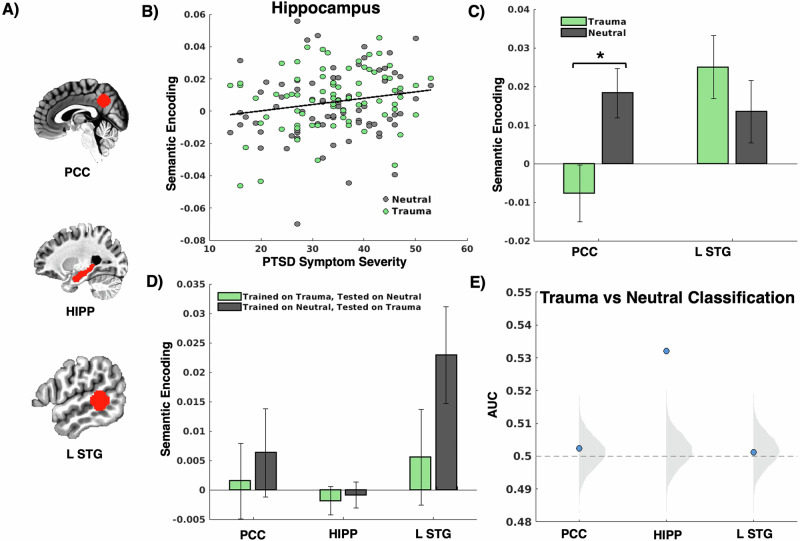
Semantic encoding as a function of PTSD symptoms and in other ROIs. **A** Depiction of the ROIs displayed in MNI space. **B** Scatter plot showing positive association between semantic encoding, regardless of narrative type, and PTSD symptom severity. **C** Mean semantic encoding for the posterior cingulate cortex (PCC) and left superior temporal gyrus (STG). The left STG demonstrated significant overall semantic encoding, and the PCC demonstrated greater semantic encoding for the neutral compared to the trauma narrative. **D** Results from cross-narrative models, in which semantic encoding models were trained on one narrative dataset (e.g., neutral) and then tested on the alternative narrative dataset (e.g., trauma). Semantic encoding of cross-trained and tested models was significant only for the left STG with no significant differences between model types. **E** SVC classification performance for decoding narrative type from patterns of voxel activity to each sentence, without considering semantic content, in each ROI. The blue dots represent the median AUC classification performance across a 10-fold cross-validation loop, with the dotted line depicting chance AUC classification performance. The gray distributions depict the permuted null distribution for that classification analysis across 10,000 iterations.

We next tested for associations between hippocampal semantic encoding and PTSD symptom severity (Fig. 4B). LMEMs, also controlling for age, education, psychiatric medication, framewise displacement, and site demonstrated positive associations between PTSD severity and semantic encoding, regardless of valence, *t*(140) = 3.56, *p* < 0.001. When repeating these LMEMs within the different hippocampal subfields, all were significantly associated with PTSD severity (*ts* > 2.9, *ps* < 0.009) except for the subiculum (*p* = 0.34; Supplemental Fig. 2).

### Semantic encoding of autobiographical memories in the PCC and STG

Semantic encoding was significant for the STG, *t*(147) = 3.22, *p* = 0.002 (Fig. 4C), with no differences between trauma and neutral narratives, *p* = 0.29, and no association with PTSD severity, *p* = 0.46. Semantic encoding was significantly greater for neutral compared to trauma narratives in the PCC, *t*(147) = −2.95, *p* = 0.004 (Fig. 4B). There was no association with PTSD severity, *p* = 0.55.

### Cross-narrative semantic encoding models

We tested whether semantic encoding models trained on one narrative type generalized to the other, unseen, narrative type. For the left STG, there was significant overall model fit, *t*(147) = 2.49, *p* = 0.013, for the cross-trained and tested models with no difference between cross-narrative models, *p* = 0.13 (Fig. 4D). However, neither the PCC nor the hippocampus cross-trained and tested models demonstrated significant model fit (*p*s > 0.5).

### Sentence activity classification analyses

We also tested if narrative type could be decoded from patterns of voxel activity to each sentence, without considering modulation by semantic content. The SVC classification analyses demonstrated significant decoding performance for only the hippocampus (median AUC = 0.53, *p* < 0.001), while neither the PCC nor the left STG differentiated narrative type (median AUCs = 0.50, *p*s > 0.56) (Fig. 4E).

### Whole-brain semantic encoding and relationship with PTSD severity

**Fig. 5:**
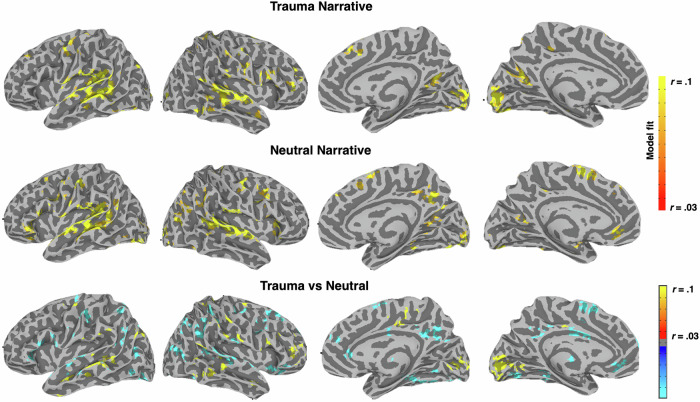
Whole-brain analysis results for the trauma narrative (top) and neutral narrative (middle) semantic encoding models. The bottom panel depicts the comparison between trauma and neutral, with warmer and cooler colors depicting greater or lesser, respectively, semantic encoding for trauma narratives.

Figure 5 displays whole-brain results of regions significantly encoding semantic content of trauma, neutral, and the contrast of trauma compared to neutral narratives (all significant clusters listed in Supplemental Tables 2–4). An additional voxelwise LMEM was conducted testing for associations between semantic encoding and PTSD severity in addition to the narrative type × PTSD severity interaction. Controlling for voxelwise comparisons, no significant clusters were detected.

## Discussion

Using our analytical approach, we detected statistically significant, yet relatively small in magnitude, hippocampal activation to semantic content for both traumatic and neutral narratives. We also demonstrated unique spatial encoding patterns of semantic content for trauma vs neutral narratives throughout the hippocampus. Specifically, voxel encoding patterns for semantic content in CA1 and the dentate gyrus modestly differentiated trauma vs neutral narratives, and semantic encoding models trained on one narrative dataset did not generalize to the other narrative dataset in the hippocampus. The degree to which the hippocampus tracked semantic content of autobiographical narratives was positively associated with PTSD symptoms. Finally, examining neurocircuitry activation to semantic content of narratives outside of just the hippocampus, the PCC and STG demonstrated differential sensitivity to semantic content of trauma vs neutral narratives. While we discuss these findings in the context of PTSD, it is relevant to note that methodology and data from this study have implications for the broader psychiatric literature where semantic memory disruptions, episodic and autobiographical memory impairments, and hippocampal abnormalities are present (e.g., schizophrenia, depression) [63–67].

Despite widespread support for hippocampal involvement in normative autobiographical memories [14–16, 50], hippocampal activity has not been reliably detected during trauma-narrative recall [9, 28, 37, 68, 69]. Indeed, a prior failure to detect hippocampal sensitivity for semantic content of trauma narratives was used to support a hypothesis that trauma memory recall was a unique cognitive entity distinct from normative autobiographical memory recall [13]. Here, we detect significant, though modest, hippocampal activity to semantic content of both traumatic and neutral narratives, and we further demonstrate that more severe PTSD symptoms are associated with *greater* sensitivity to autobiographical narrative semantic content regardless of narrative type. We suspect our methodology—which both captured moment-by-moment nuances of the autobiographical narratives and included a substantially larger sample size compared to several prior PTSD script-driven imagery studies [9, 13, 28, 68, 69]—enabled better sensitivity to detecting trauma narrative-related neurocircuitry patterns. Additionally, the decoding analyses also demonstrated unique patterns of hippocampal activity when merely listening to the trauma vs neutral narratives. Combined, these data demonstrate hippocampal engagement during trauma-narrative recall with respect to both recalling the narrative, per se, as well as in response to the specific semantic content of individual sentences. These findings suggest a neurobiological mechanism engaged during therapies that ask patients to repeatedly recall the trauma narrative [25], such that this clinically efficacious procedure appears to be actively engaging the hippocampus, albeit modestly, and thereby enabling extinction learning [70, 71] by re-contextualizing the complex trauma narrative [72, 73].

While we demonstrate statistically significant hippocampal activity towards semantic content of trauma narratives, we also demonstrate that the manner by which the hippocampus tracks semantic content differs between narrative types. Specifically, spatial patterns encoding semantic content across the entire hippocampus were uncorrelated between trauma and neutral narratives, and spatial encoding patterns of semantic content in CA1 and the dentate gyrus could significantly, though modestly, discriminate between trauma and neutral narratives. Further, the semantic encoding models trained on one narrative dataset did not generalize to the other dataset in the hippocampus. Accordingly, there does appear to be narrative specific sensitivity to precisely how the hippocampus tunes towards semantic content. The dentate gyrus has been implicated in several cognitive functions [74, 75], including pattern separation, novelty detection, indexing, and temporal tagging. The CA1 has been implicated in autobiographical memory [76] and fear extinction learning [57, 77] and gray matter volumes are reduced in PTSD [56]. Nonetheless, the lack of a non-traumatic negative valence script control precludes inferences regarding trauma-narrative specific differences vs more general differences due to negative valence.

Beyond just the hippocampus, we also tested the role of the PCC and left STG as specific regions-of-interest and conducted a voxelwise analysis. The left STG is canonically associated with language and prior natural language processing fMRI research using semantic embeddings from large language models has found prominent semantic encoding in the left STG [44, 52]. Here, we similarly identified statistically significant, though modest, semantic encoding in the left STG for both narrative types, as the cross-narrative models demonstrated generalized semantic encoding in the left STG, and the voxelwise test demonstrated significantly greater semantic encoding for trauma narratives in a portion of the left STG. Concurrently, narrative type could not be decoded with sentence-by-sentence activity patterns, irrespective of semantic content, in the STG. These data suggest heightened sensitivity specifically to semantic content of trauma, compared to neutral, autobiographical narratives in canonical language processing circuits. By contrast, we observed decreased semantic encoding of trauma compared to neutral narratives in the PCC, and the voxelwise analysis additionally suggested decreased encoding in the medial PFC. The PCC and mPFC are major hubs of the default mode network (DMN), and in addition to subserving self-directed mentation, a wealth of data supports a role of the DMN in autobiographical memory [53, 78, 79] and intrinsically-oriented cognition [80–82], with research suggesting that DMN activity decreases upon attention to external stimuli [53]. One explanation for the heightened STG semantic encoding, yet decreased PCC and mPFC semantic encoding, during trauma memory recall is that the trauma-narrative audio and visual text operated as more salient external stimuli and thereby suppressed DMN semantic encoding. There was no relationship between PTSD symptoms and either the PCC or left STG, nor were any significant symptom-semantic encoding relationships detected in the whole-brain analysis. Again, the lack of a non-traumatic negative valence narrative control precludes inferences regarding trauma-specific differences vs more general negative valence effects.

The study has some limitations. One important limitation is that the trauma versus neutral contrasts are intrinsically confounded with a fixed presentation order and with differences in emotional valence/arousal, and as a result, any claims about trauma-specific spatial pattern differences should be considered tentative and in need of confirmation in future studies with randomized order and negative, non-trauma control narratives. Our sample was restricted to women with PTSD related to interpersonal violence, and the inclusion of only individuals with PTSD also means we could only detect associations with PTSD symptom severity above a diagnostic threshold. It is also important to note that the relative magnitude of effects were modest: model fit values for the hippocampus were small, classification performance was only modest, and associations with PTSD severity were of moderate effect sizes. Finally, future work will also benefit from higher-resolution imaging to more precisely characterize hippocampal subfields.

## Supplementary information

supplement

## Data Availability

De-identified data and code have been deposited at Open Science Framework and are publicly available as of the date of publication: 10.17605/OSF.IO/U5BEA. Any additional information required to reanalyze the data reported in this paper is available from the lead contact upon request.
