# Supplementary material for: Semantic encoding of trauma memories in the hippocampus among individuals with PTSD
Source: Neuropsychopharmacology. 2026 Apr 14;51(9):1690–8. doi: 10.1038/s41386-026-02402-5 (PMC13388956; doi:10.1038/s41386-026-02402-5)
Supplement: Supplementary file 1 — supplement [file 41386_2026_2402_MOESM1_ESM.docx]

**Supplementary Methods**

**Autobiographical Narrative Task**

The task used here was modeled after our prior study^1^, which is an adaptation of the commonly used script-driven imagery ^2–5^. Two scripts, one detailing a low arousing and emotionally neutral memory and one detailing the individual’s index traumatic event, were created through a collaborative interview between participants and a research staff member. Following standardized instructions provided by research staff, the participant described the event aloud with the research staff transcribing. The research staff prompted to ensure the narrative focused on a specific event that occurred within a 24-hour period (e.g., as opposed to something that unfolded over days or weeks) and included sensory, physiological, and emotional detail. Traumatic memory scripts aimed to induce a 7/10 level of distress. The research staff worked with participants to add or remove details if the participant’s distress rating was higher or lower than a 7/10. Each script ranged in length between 330-350 words. Each script was audio recorded by a female team member to whom the participant was naïve. Each script audio recording had a duration between 2 minutes 20 seconds to 2 minutes 30 seconds in length. Speech rate and loudness were controlled experimentally, such that all scripts were constrained to the same audio length and the staff providing the audio recordings were trained to speak at a certain rate. The audio recordings were then edited to have matched loudness using audio editing software. Audio recordings were all reviewed by an independent staff member to confirm length, speech rate, neutral prosody, and word pronunciation. Deviations in the audio recordings detected by the reviewer resulted in the audio being re-recorded until it met the requirements. The audio recordings were finally edited to have matched loudness using audio editing software.

During scanning, audio recordings of each script were accompanied by images of text of the script depicted with white font against a black background. This allowed the participant to read along as they listened to their memory. Each memory was presented four times in a row, with the neutral memory always occurring first. The repeated presentation thereby modeled imaginal exposure for PTSD^1,6^ and allowed for characterization of changes in neurocircuitry mechanisms across repeated presentations. Each run started with 25 s of a fixation cross, then a 5 s cue letting participants know the script was about to start. Participants provided self-report ratings of anxiety, vividness of the memory, and dissociation on 0-10 Likert scales after each script repetition. As indicated in supplemental Figure 1, self-reported anxiety and dissociation, but not vividness, was higher during the trauma versus neutral memory presentations.

**MRI acquisition and image preprocessing**

At the University of Wisconsin Madison site, fMRI data were acquired on a GE MR750 3T scanner (GE Healthcare, Chicago, IL, USA) using a Nova 32-channel head coil. T1-weighted anatomic images were acquired with a MP-RAGE sequence (matrix = 256x256, 156 axial slices, TR/TE/FA = 8.2ms/3.2ms/12°, FOV = 25.6cm, final resolution = 1x1x1mm). A multiband sequence, in close approximation to the ABCD protocol, was used to collect the functional images with the following parameters: TR/TE/FA = 800ms/25ms/65°, FOV = 21.6cm, matrix = 90 x 90, 54 axial slices, slice thickness = 2.4mm, acceleration factor = 6.

At the University of Texas at Austin site, fMRI data were acquired on a Siemens VIDA 3T scanner (Siemens, Erlangen, Germany) using a 64-channel head coil. T1-weighted anatomic images were acquired with a MP-RAGE sequence (matrix = 256x256, 208 sagittal slices, TR/TE/FA = 2400ms/2.18ms/8°, FOV = 25.6cm, final resolution = .8x.8x.8mm). A multiband sequence, in close approximation to the ABCD protocol, was used to collect the functional images with the following parameters: TR/TE/FA = 788ms/30ms/52°, FOV = 21.5cm, matrix = 90 x 90, 60 axial slices, slice thickness = 2.5mm, acceleration factor = 6.

Image preprocessing followed standard steps and was completed using AFNI software. Images first underwent either field map correction (at the UW site) or distortion correction (at UT site) using a 10 TR reversed phase encoding direction scan after each functional run (ie.., P > A rather than A > P during the functional scans). Images then underwent despiking, slice timing correction, deobliquing, motion correction using rigid body alignment, and alignment to participant’s normalized anatomical images (3dQwarp and 3dNwarpApply). Following recommendations^7–9^, we corrected for head motion related signal artifacts by using motion regressors derived from Volterra expansion, consisting of [R R^2^ R_t-1_ R^2^_t-1_], where R refers to each of the 6 motion parameters, and separate regressors for mean signal in the CSF and WM. Images then underwent spatial smoothing using a 8 mm FWHM Gaussian filter (AFNIs 3dBlurToFWHM that estimates the amount of smoothing to add to each dataset to result in the desired level of final smoothing), detrending, low frequency (128s) bandpass filtering, and rescaling into percent signal change. Images were normalized using the MNI 152 template brain. Additionally, we censored TRs from the first-level GLMs based on threshold of framewise displacement (FD) > 0.4. FD refers to the sum of the absolute value of temporal differences across the 6 motion parameters; thus, a cut-off of 0.4 results in censoring TRs where the participant moved, in total across the 6 parameters, more than ~0.4 mm plus the immediately following TR (to account for delayed effects of motion artifact). Additionally, we censored isolated TRs where the preceding and following TRs were censored, and we censored entire runs if more than 50% of TRs within that run were censored. This led to the removal of 1 PTSD participant from the trauma memory recall task.

**Regions of interest**

The hippocampus was defined from the Harvard Oxford atlas. Hippocampal subfields CA1, CA2, CA3, dentate gyrus, and subiculum were defined from the Julich atlas[63] available in AFNI. The posterior cingulate cortex (PCC) was defined as a 10mm sphere centered on the peak PCC coordinates of a Neurosynth[64] meta-analysis for the search term Default Mode. The left superior temporal gyrus (STG) was similarly defined as a 10mm sphere centered on the peak left STG coordinates of a Neurosynth[64] meta-analysis for the search term Language.

**Script type lexical property comparisons**

Mean word length for trauma and neutral scripts, respectively, was 3.95 (.17) and 3.93 (.19), and the comparison is non-significant: *t*=.11, p = .91. Mean sentence length for trauma and neutral scripts, respectively, was 14.83 (4.94) and 14.76 (3.53), and the comparison is non-significant: *t*=.56, p = .58. Mean number of sentences for trauma and neutral scripts, respectively, was 24.86 (7.86) and 24.22 (8.25), and the comparison is non-significant: *t*=.63, p = .52.

Speech rate and loudness were controlled experimentally, such that all scripts were constrained to the same audio length and the staff providing the audio recordings were trained to speak at a certain rate. The audio recordings were then edited to have matched loudness using audio editing software.

**Semantic content differentiates trauma and neutral autobiographical narratives.**

As validation of the sentence transformer methodology, we examined similarity of semantic embeddings for trauma and neutral sentences. Cosine similarity was calculated between each sentence pair (Supplemental Figure 2A) and compared within- and between-memory types (Supplemental Figure 2B). Within-memory type sentence similarity was greater than between sentence-similarity, *p*s < .001. Semantic similarity among trauma-trauma pairs was also greater than among neutral-neutral pairs, *p* < .001, consistent with trauma memories sharing a common semantic theme (interpersonal violence) and neutral memories lacking an instructed semantic theme. A support vector classifier (SVC) trained on the semantic embeddings could differentiate trauma from neutral sentences (10-fold cross-validation mediation AUC = .98; Supplemental Figure 2C). While the separation of trauma and neutral semantic embeddings is visually apparent following dimension reduction (Supplemental Figure 2D), the overlap between script types and variability in within-vs-between script sentence similarity is also apparent.

| **Table 1. Demographic and Clinical Characteristics** | |
| --- | --- |
| **Variable** | **n = 79** |
| Age (years) | 30.5 (8.4) |
| Education (years) | 15.8 (2.3) |
| Sex Assigned at Birth |  |
| Female (%) | 100 |
|  |  |
| Ethnicity | |
| White (%) | 72.2 |
| Black (%) | 6.3 |
| Asian (%) | 2.5 |
| Hispanic/Latino (%) | 11.4 |
| Mixed Race/Other (%) | 7.6 |
|  |  |
| IQ | |
| Vocab T Score | 59.0 (9.5) |
| Matrix Reasoning T Score | 50.7 (6.3) |
| FSIQ Score | 108.5 (11.2) |
| Direct Assault Types (#) | 4.2 (2.4) |
| Sexual Assault (%) | 88.6 |
| Physical Assault (%) | 70.9 |
| Physical Abuse (%) | 59.5 |
| Age first assault (years) | 9.7 (6.6) |
| Age last assault (years) | 24.7 (8.1) |
| Time since last assault (years) | 5.9 (6.4) |
| Current mood disorders (%) | 44.3 |
| Current co-morbid anxiety disorders (%) | 49.4 |
| Current GAD (%) | 28.8 |
| Current OCD (%) | 16.5 |
| CAPS-V Total Severity Score | 33.9 (9.4) |
| CAPS Cluster B Severity Score | 9.1 (2.9) |
| CAPS Cluster C Severity Score | 4.2 (1.5) |
| CAPS Cluster D Severity Score | 12.4 (4.3) |
| CAPS Cluster E Severity Score | 8.6 (3.4) |
|  |  |

Note. IQ was assessed using the Wechsler Abbreviated Scale of Intelligence Second Edition using the Verbal Items module and Matrix Reasoning module. CAPS Clinician Administered PTSD Scale DSM-V was used to assess PTSD. The Diamond was used to assess the following disorders: Obsessive Compulsive Disorder (OCD), Social Anxiety Disorder, Panic Disorder, Agoraphobia, Generalized Anxiety Disorder (GAD), Bipolar I and Bipolar II, Major Depressive Disorder, Anorexia Nervosa, Bulimia Nervosa, Binge-Eating Disorder, and Substance Use Disorder.  NSA Trauma Assessment assessed assault exposures.

**Supplementary Results**

| **Narrative** | **Region** | **MNI center-of-mass coordinates** | | | **Peak *r*** | **Cluster size** |
| --- | --- | --- | --- | --- | --- | --- |
|  |  | **X** | **Y** | **Z** |  |  |
| Trauma | Left superior temporal gyrus | 58 | 26 | 5 | .20 | 1309 |
|  | Left visual cortex | 0 | 90 | -5 | .11 | 662 |
|  | Right superior temporal gyrus | -59 | 21 | 2 | .17 | 640 |
|  | Left middle frontal gyrus | 50 | 0 | 51 | .10 | 108 |
|  | Right inferior frontal gyrus | -54 | -18 | 6 | .08 | 88 |
|  | Right superior frontal gyrus | -13 | -17 | 63 | .10 | 71 |
|  | Left posterior cingulate cortex | 13 | 67 | 25 | .06 | 70 |
|  | Right visual cortex | -29 | 89 | 2 | .10 | 65 |
|  | Right middle frontal gyrus | -29 | -53 | 19 | .07 | 50 |
|  | Left cuneus gyrus | 38 | 80 | 36 | .08 | 50 |
|  | Right superior parietal lobule | -20 | 38 | 66 | .10 | 50 |
|  | Right inferior temporal gyrus | -58 | 50 | -13 | .07 | 47 |
|  | Right posterior cingulate cortex | -23 | 60 | 21 | .08 | 47 |
|  | Right rostral anterior cingulate | -4 | -28 | 44 | .05 | 46 |
|  | Left visual cortex | 27 | 92 | 4 | .08 | 40 |
|  | Right inferior frontal gyrus | -64 | 18 | 28 | .07 | 36 |
|  | Right middle frontal gyrus | -55 | 2 | 44 | .10 | 36 |
|  | Right middle frontal gyrus | -41 | -43 | 3 | .07 | 35 |
|  | Left posterior cingulate cortex | 13 | 60 | 13 | .05 | 35 |
|  | Left visual cortex | 43 | 79 | -17 | .07 | 27 |
|  | Right posterior cingulate cortex | -13 | 57 | 19 | .06 | 26 |
|  | Right precentral gyrus | -60 | 3 | 24 | .05 | 26 |
|  | Right middle temporal gyrus | -48 | -1 | -25 | .06 | 24 |
|  | Left middle frontal gyrus | 24 | -39 | 37 | .05 | 24 |
|  | Right middle frontal gyrus | -36 | -15 | -57 | .07 | 24 |
|  | Right superior temporal gyrus | -60 | 44 | 14 | .06 | 23 |
|  | Left middle temporal gyrus | 52 | 5 | -22 | .06 | 22 |
|  | Right middle frontal gyrus | -35 | -54 | 8 | .05 | 22 |
|  | Left precentral gyrus | 54 | 9 | 39 | .09 | 22 |
|  | Right cerebellar cortex | -21 | 61 | -25 | .06 | 21 |
|  | Left visual cortex | 48 | 63 | -21 | .06 | 21 |
|  | Right posterior cingulate cortex | -10 | 55 | 8 | .06 | 21 |
|  | Left precuneus gyrus | 7 | 65 | 58 | .06 | 21 |

**Supplementary Table 2. Results of voxelwise test of semantic encoding for trauma narratives.**

| **Narrative** | **Region** | **MNI center-of-mass coordinates** | | | **Peak *r*** | **Cluster size** |
| --- | --- | --- | --- | --- | --- | --- |
|  |  | **X** | **Y** | **Z** |  |  |
| Neutral | Left superior temporal gyrus | 60 | 21 | 4 | .21 | 1186 |
|  | Left superior temporal gyrus | -60 | 20 | 4 | .16 | 737 |
|  | Right visual cortex | -20 | 87 | -13 | .10 | 238 |
|  | Left visual cortex | 26 | 87 | -15 | .11 | 205 |
|  | Right superior frontal gyrus | 1 | -13 | 60 | .11 | 182 |
|  | Inferior temporal gyrus | 46 | 55 | -21 | .07 | 149 |
|  | Left middle frontal gyrus | 53 | 7 | 45 | .13 | 142 |
|  | Right middle frontal gyrus | -42 | -15 | 27 | .08 | 118 |
|  | Right visual cortex | -23 | 95 | 9 | .09 | 104 |
|  | Right middle frontal gyrus | -52 | 1 | 45 | .09 | 92 |
|  | Left superior temporal gyrus | 52 | 47 | 9 | .10 | 83 |
|  | Right visual cortex | -46 | 73 | 15 | .07 | 75 |
|  | Right posterior cingulate cortex | -7 | 55 | 38 | .07 | 68 |
|  | Left inferior frontal gyrus | 49 | -31 | -8 | .07 | 63 |
|  | Left middle frontal gyrus | 45 | -23 | 25 | .07 | 63 |
|  | Right posterior cingulate cortex | -17 | 61 | 28 | .06 | 58 |
|  | Left inferior frontal gyrus | 54 | -19 | 9 | .07 | 51 |
|  | Right inferior frontal gyrus | -49 | -23 | -5 | .06 | 47 |
|  | Right insula | -31 | -28 | -2 | .06 | 45 |
|  | Right supramarginal gyrus | -54 | 45 | 35 | .05 | 38 |
|  | Right visual cortex | -46 | 63 | 24 | .05 | 36 |
|  | Left superior parietal lobule | 20 | 68 | 60 | .06 | 32 |
|  | Left inferior frontal gyrus | 63 | 0 | 20 | .08 | 31 |
|  | Left superior temporal gyrus | 56 | 56 | 21 | .07 | 29 |
|  | Right lingual gyrus | -27 | 39 | -15 | .07 | 26 |
|  | Right precuneus gyrus | -7 | 64 | 21 | .06 | 26 |
|  | Right caudate nucleus | -14 | -4 | 18 | .06 | 25 |
|  | Left superior frontal gyrus | 6 | -42 | 37 | .06 | 25 |
|  | Left middle frontal gyrus | 39 | -1 | 62 | .10 | 25 |
|  | Left cerebellar cortex | 20 | 61 | -24 | .08 | 24 |
|  | Right inferior temporal gyrus | -43 | 58 | -18 | .06 | 24 |
|  | Right inferior frontal gyrus | -62 | -2 | 20 | .07 | 24 |
|  | Left posterior cingulate cortex | 11 | 59 | 11 | .05 | 23 |
|  | Left cerebellar cortex | 3 | 60 | -50 | .06 | 21 |
|  | Left rostral anterior cingulate | 10 | -38 | -10 | .06 | 21 |
|  | Left superior parietal lobule | 30 | 63 | 56 | .06 | 21 |

**Supplementary Table 3. Results of voxelwise test of semantic encoding for neutral narratives.**

| **Contrast** | **Region** | **MNI center-of-mass coordinates** | | | **Peak *r*** | **Cluster size** |
| --- | --- | --- | --- | --- | --- | --- |
|  |  | **X** | **Y** | **Z** |  |  |
| Trauma  vs Neutral | Left visual cortex | -1 | 90 | 3 | .12 | 248 |
|  | Left superior frontal gyrus | 2 | -13 | 60 | -.13 | 145 |
|  | Right superior parietal lobule | -50 | 47 | 31 | -.10 | 122 |
|  | Left superior temporal gyrus | 65 | 32 | 0 | .10 | 118 |
|  | Right visual cortex | -47 | 76 | 14 | -.10 | 109 |
|  | Right posterior cingulate cortex | -5 | 48 | 35 | -.09 | 109 |
|  | Right middle frontal gyrus | -39 | -50 | 5 | .13 | 107 |
|  | Right precentral gyrus | -64 | 18 | 31 | .10 | 101 |
|  | Left lingual gyrus | 21 | 75 | -11 | -.10 | 98 |
|  | Right superior temporal gyrus | -65 | 22 | 8 | -.10 | 89 |
|  | Right precuneus gyrus | -34 | 40 | 49 | -.10 | 76 |
|  | Right superior frontal gyrus | -18 | -37 | 50 | -.10 | 64 |
|  | Right visual cortex | -22 | 86 | -17 | -.09 | 62 |
|  | Left precentral gyrus | 53 | 11 | 45 | -.12 | 62 |
|  | Right middle frontal gyrus | -42 | -14 | 26 | -.10 | 54 |
|  | Left precuneus gyrus | 31 | 76 | 45 | -.11 | 54 |
|  | Left cerebellar cortex | 4 | 59 | -48 | -.11 | 45 |
|  | Left superior temporal gyrus | 59 | 16 | 3 | -.10 | 44 |
|  | Left middle temporal gyrus | 64 | 7 | -13 | -.09 | 43 |
|  | Right lingual gyrus | -28 | 38 | -15 | -.09 | 40 |
|  | Right superior temporal gyrus | -61 | 52 | 11 | .11 | 40 |
|  | Left visual cortex | 7 | 87 | -16 | .10 | 39 |
|  | Left insula | 42 | 10 | -10 | -.08 | 39 |
|  | Right lingual gyrus | -31 | 50 | -18 | -.09 | 38 |
|  | Right middle frontal gyrus | -26 | -24 | 43 | -.09 | 38 |
|  | Left lingual gyrus | 28 | 42 | -14 | -.09 | 37 |
|  | Left superior temporal gyrus | 69 | 45 | 4 | -.14 | 37 |
|  | Right cerebellar cortex | -12 | 68 | -15 | -.09 | 36 |
|  | Cerebellar vermis | 0 | 77 | -24 | -.09 | 35 |
|  | Right middle temporal gyrus | -47 | 42 | 10 | -.09 | 35 |
|  | Left cerebellar cortex | 46 | 52 | -29 | -.09 | 32 |
|  | Left inferior frontal gyrus | 64 | 37 | 26 | .11 | 32 |
|  | Left superior parietal lobule | 38 | 51 | 55 | -.10 | 32 |
|  | Left inferior frontal gyrus | 46 | -29 | -5 | -.10 | 31 |
|  | Left precuneus gyrus | 35 | 81 | 37 | .10 | 31 |
|  | Right superior temporal gyrus | -58 | -9 | -9 | -.11 | 30 |
|  | Left inferior frontal gyrus | 59 | -16 | 9 | -.09 | 30 |
|  | Left middle frontal gyrus | 46 | 0 | 55 | .10 | 30 |
|  | Right inferior frontal gyrus | -47 | -27 | -13 | -.08 | 28 |
|  | Right post central gyrus | -21 | 39 | 66 | .09 | 28 |
|  | Right superior temporal gyrus | -54 | 29 | -3 | .08 | 25 |
|  | Left superior frontal gyrus | 8 | -68 | 15 | -.11 | 25 |
|  | Left middle frontal gyrus | 63 | -3 | 17 | -.09 | 25 |
|  | Left supramarginal gyrus | 55 | 70 | 29 | .08 | 25 |
|  | Right fusiform gyrus | -41 | 58 | -18 | -.09 | 24 |
|  | Right inferior frontal gyrus | -25 | -31 | -16 | -.09 | 24 |
|  | Left superior parietal lobule | 31 | 57 | 63 | -.08 | 24 |
|  | Left superior parietal lobule | 19 | 64 | 67 | -.08 | 24 |
|  | Left superior frontal gyrus | 2 | 4 | 70 | -.11 | 24 |
|  | Right inferior temporal gyrus | -59 | 49 | -12 | .07 | 23 |
|  | Right inferior frontal gyrus | -58 | -16 | 8 | .09 | 23 |
|  | Right posterior cingulate cortex | -14 | 63 | 33 | -.08 | 23 |
|  | Left middle temporal gyrus | 49 | -5 | -41 | .08 | 22 |
|  | Left visual cortex | 32 | 84 | -17 | -.09 | 22 |
|  | Left superior temporal gyrus | 62 | 21 | 10 | .11 | 22 |
|  | Left anterior cingulate cortex | 4 | 1 | 30 | -.08 | 22 |
|  | Right superior temporal gyrus | -40 | -24 | -30 | -.09 | 21 |
|  | Left thalamus | 5 | 12 | 3 | -.07 | 21 |
|  | Right superior frontal gyrus | -6 | -50 | 16 | -.08 | 21 |
|  | Right posterior cortex | -2 | 16 | 43 | .07 | 21 |
|  | Right precentral gyrus | -49 | 0 | 52 | -.08 | 21 |

**Supplementary Table 4. Results of voxelwise test of semantic encoding for the contrast of trauma vs neutral narratives.**


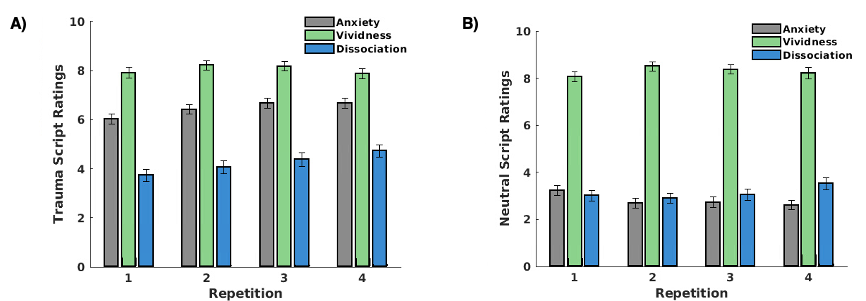


**Supplemental Figure 1. Anxiety, vividness, and dissociation ratings for the trauma and neutral autobiographical narrative scripts.**


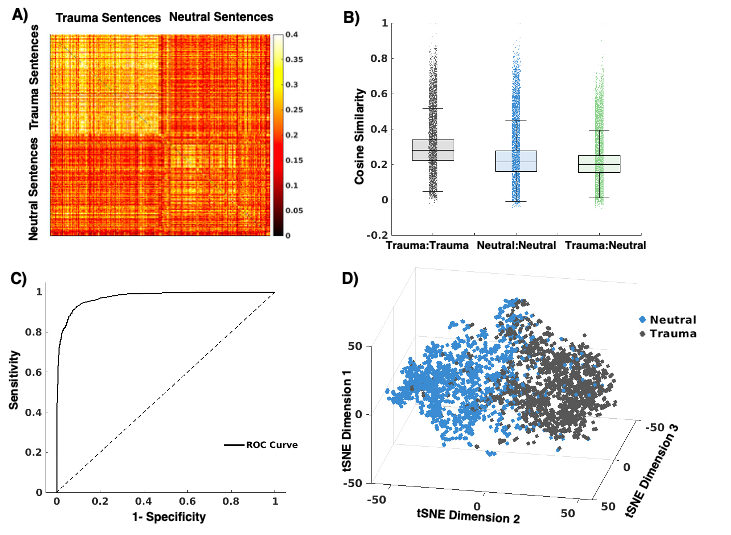


**Supplemental Figure 2**. **A)** Heat map depicting cosine similarity between each sentence’s semantic embedding, sorted by script type. **B)** Boxplots indicating mean and distributions of cosine similarity with script types and between script types. **C)** Receiver operator characteristics of a support vector classifier (SVC) trained to differentiate trauma vs neutral sentences based on semantic embeddings. The SVC used a 10-fold cross-validation procedure. **D)** 3D scatterplot indicating separation of semantic embeddings between script types. The three dimensions are plotted following data reduction with t-distributed stochastic neighbor embedding (t-SNE).


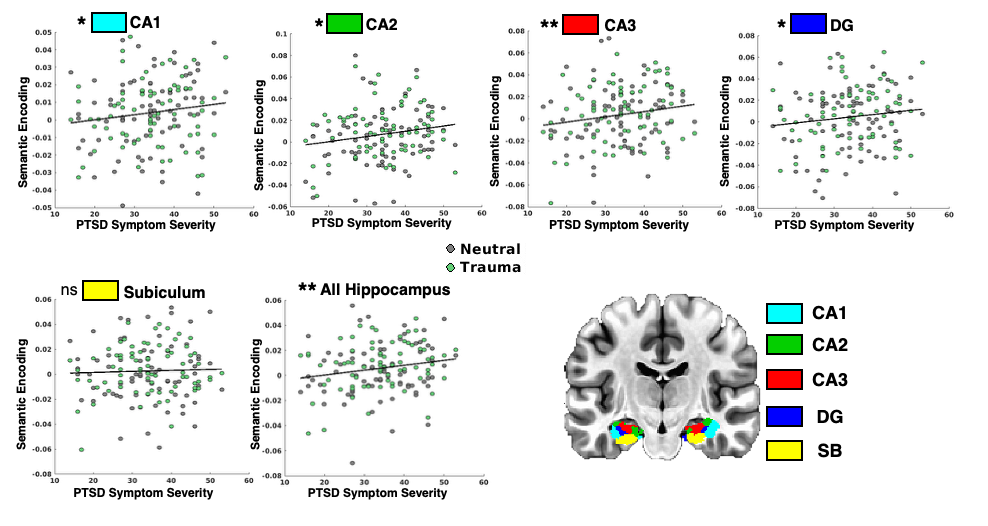


**Supplemental Figure 3. Scatterplots depicting scalar relationships between semantic embedding in the different hippocampal subfields and PTSD symptom severity.** All relationships are signification except for the subiculum.
